# Bonding brackets on white spot lesions pretreated by means of two methods

**DOI:** 10.1590/2177-6709.21.2.039-044.oar

**Published:** 2016

**Authors:** Julia Sotero Vianna, Mariana Marquezan, Thiago Chon Leon Lau, Eduardo Franzotti Sant'Anna

**Affiliations:** 1 Private practice, Rio de Janeiro, Rio de Janeiro, Brazil.; 2 Postdoc resident, Universidade Federal do Rio de Janeiro (UFRJ), Department of Pediatric Dentistry and Orthodontics, Rio de Janeiro, Rio de Janeiro, Brazil. Dentist, Universidade Federal de Santa Maria (UFSM), Department of Restorative Dentistry, Santa Maria, Rio Grande do Sul, Brazil.; 3 PhD resident, Universidade Federal do Rio de Janeiro (UFRJ), Rio de Janeiro, Rio de Janeiro, Brazil.; 4 Professor, Universidade Federal do Rio de Janeiro (UFRJ), Rio de Janeiro, Rio de Janeiro, Brazil.

**Keywords:** Dental caries, Orthodontic brackets, Shear bond strength.

## Abstract

**Objective:**

The aim of this study was to evaluate the shear bond strength (SBS) of brackets bonded to demineralized enamel pretreated with low viscosity Icon Infiltrant resin (DMG) and glass ionomer cement (Clinpro XT Varnish, 3M Unitek) with and without aging.

**Methods:**

A total of 75 bovine enamel specimens were allocated into five groups (n = 15). Group 1 was the control group in which the enamel surface was not demineralized. In the other four groups, the surfaces were submitted to cariogenic challenge and white spot lesions were treated. Groups 2 and 3 were treated with Icon Infiltrant resin; Groups 4 and 5, with Clinpro XT Varnish. After treatment, Groups 3 and 5 were artificially aged. Brackets were bonded with Transbond XT adhesive system and SBS was evaluated by means of a universal testing machine. Statistical analysis was performed by one-way analysis of variance followed by Tukey post-hoc test.

**Results:**

All groups tested presented shear bond strengths similar to or higher than the control group. Specimens of Group 4 had significantly higher shear bond strength values (*p* < 0.05) than the others.

**Conclusion:**

Pretreatment of white spot lesions, with or without aging, did not decrease the SBS of brackets.

## resumo

**Introdução:**

o objetivo do presente estudo foi avaliar a resistência ao cisalhamento de braquetes colados ao esmalte desmineralizado e pré-tratado com uma resina infiltrante de baixa viscosidade (Icon, DMG) ou com um selante ionomérico (Clinpro XT Varnish, 3M Unitek), com e sem envelhecimento.

**Métodos:**

setenta e cinco (75) amostras de esmalte bovino foram divididas em cinco grupos (n = 15). No Grupo 1 (controle), a superfície do esmalte não foi desmineralizada. Nos outros quatro grupos, as superfícies foram submetidas ao desafio cariogênico e as lesões de manchas brancas foram tratadas previamente à colagem de braquetes. Nos Grupos 2 e 3, as lesões foram tratadas com a resina Icon; nos Grupos 4 e 5, com o selante Clinpro XT Varnish. Após o tratamento, os Grupos 3 e 5 foram envelhecidos artificialmente. Os braquetes foram colados, sobre as lesões tratadas, utilizando-se o sistema adesivo Transbond XT, e a resistência ao cisalhamento foi avaliada em máquina universal de ensaios. A análise estatística foi realizada por meio da análise de variância e, posteriormente, pelo teste *post-hoc* de Turkey.

**Resultados:**

todos os grupos testados apresentaram resistência ao cisalhamento semelhante ou maior do que o grupo controle. As amostras do Grupo 4 apresentaram valores de resistência ao cisalhamento significativamente maiores (*p* < 0,05) do que as dos demais grupos.

**Conclusão:**

o tratamento das manchas brancas, com ou sem envelhecimento, não diminuiu a resistência ao cisalhamento dos braquetes.

## INTRODUCTION

White spot lesions around orthodontic brackets are a clinical problem arising during and after orthodontic treatment.^1^ They are associated with plaque accumulation due to increased difficulty maintaining oral hygiene in the presence of orthodontic appliances, and modification of the quantity and quality of oral microbiota after appliance placement.^2^ It is believed that approximately 50% of orthodontic patients develop these lesions,^3^ and several studies have reported a significant increase in the prevalence and severity of tooth demineralization in patients during orthodontic treatment.^4^ ^-^ ^11^

The traditional method for treating white spots is to remineralize incipient lesions with the use of fluorides.^12^ The remineralization process involves diffusion of calcium and phosphate ions into the subsurface lesion to restore the lost tooth structure.^13^ Fluoride helps to reharden the softened tooth structure by increasing the percentage of mineral deposition, but there is controversy about whether this treatment improves the milky color of the porous enamel, or whether it only rehardens the surface layer with less effect on its appearance.^14^ Treatment modalities aimed to mask white spots and render them less visible have been developed over the years.^15^ ^-^ ^18^ The most recent treatment modality is based on the application of low viscosity inflitrant resins to the affected area.

Rebonding brackets is a common procedure during orthodontic treatment^19^ because of the high debonding rates of orthodontic brackets resulting from mechanical and thermal stresses, or because orthodontists need to change the position of brackets.^20^ White spot lesions may be found on the buccal surface of teeth during rebonding. This would lead the orthodontist to being concerned about the adequacy of the bonding procedure to these surfaces, and only a few studies have demonstrated the influence of white spot lesion treatment on the shear bond strength of brackets.^20^ ^-^ ^23^

The need for bonding brackets to surfaces treated with fluoride material or infiltrant resins may also arise in cases of orthodontic retreatment. The influence of the time elapsed between white spot lesion treatment and the bonding procedure has not been previously investigated.

The purpose of this *in vitro* study was to evaluate the shear bond strength of brackets bonded to demineralized and pretreated enamel surfaces immediately after white spot lesion treatment and after aging procedure.

## MATERIAL AND METHODS

A total of 75 bovine incisor crowns were debrided, rinsed and stored in distilled water with 0.1% thymol to prevent dehydration and bacterial growth. Crowns were refrigerated at 5 °C before the experiment. The teeth were embedded in cylindrical molds (25 mm in diameter, Tigre, Joinville, Brazil). The labial surfaces of the crowns were pressed against a glass plate, and the molds were filled with self-curing acrylic resin (JET-Classic, Campo Limpo, Brazil). The enamel surfaces were ground flat and progressively polished with 400, 600 and 1200 grit abrasive papers (Polishing Machine model PLF-Fortel industry and trade LTDA, São Paulo/SP, Brazil) to obtain a flat surface. Afterwards the specimens were stored in distilled water.

**Figure 1:**
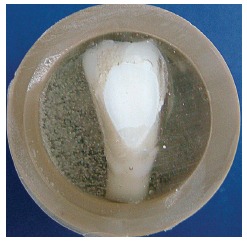
Aspect of the enamel after cariogenic challenge. Compare white spot lesion with healthy enamel (lower portion) previously protected with nail polish.

The samples were randomly assigned into five groups (n = 15, calculated by one-tailed Student's-t test with 90% power and 5% significance level). In Group 1 (control), the enamel surface was not demineralized. In the other four groups, the surfaces were submitted to cariogenic challenge, and white spot lesions were treated. The specimens received a mask to standardize the demineralization area. The remaining tooth surfaces were covered with acid-resistant varnish (nail varnish).^24^ A demineralizing solution (0.05 mol/L acetate buffer, pH 5.0, containing 1.28 mmol/L Ca, 0.74 mmol/L Pi and 0.03 µg F/mL)^25^ ^,26^ was used to induce caries-like lesions on enamel. The teeth were subjected to the pH-cycling caries reversal model.^25^ ^,26^ The aspect of the white spot lesions may be observed in Figure 1.

After demineralization, the specimens were stored in artificial saliva at 37 °C for 56 hours.^20^ The composition of artificial saliva was hydrogen carbonate (22.1 mmol/L), potassium (16.1 mmol/L), sodium (14.1 mmol/L), hydrogen phosphate (2.6 mmol/L), boric acid (0.8 mmol/L), calcium (0.7 mmol/L), thiocyanate (0.4 mmol/L) and magnesium (0.2 mmol/L), with pH ranging between 7.4 and 7.8.^29^ Artificial saliva may have induced some degree of remineralization of the lesions by incorporation and precipitation of minerals^12^ during this time interval, which may also happen to white spots in a natural oral environment.

**Figure 2:**
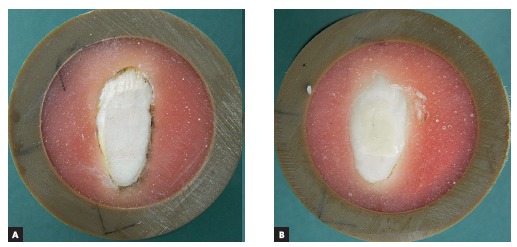
Enamel treated with Icon infiltration treatment (A) and ClinproXT Varnish (B).

White spot lesions were treated in Groups 2 and 3, using Icon (DMG, Hamburg, Germany), a low viscosity infiltrant resin (Fig 2A). Groups 4 and 5 were treated with Clinpro XT Varnish (3M Unitek, Landsberg, Germany), a glass ionomer cement (Fig 2B). Pretreatment and application of products were performed according to the respective manufacturers' instructions (Table 1).

**Figure 3:**
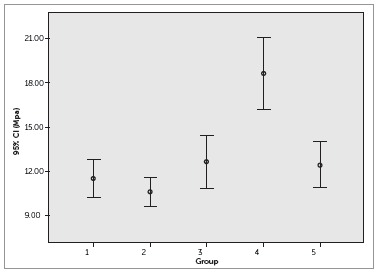
Box plot for results of shear bond strength.

**Table 1:** Groups, material and application protocols used in the present experiment.

Group	n	Material	Manufacturer	Composition	Lot #	Application	Aging
1	15	-	-	-	-	-	No
2	15	Icon	DMG (Hamburg, Germany)	Icon-Etch: hydrochloric acid salicylic pyrogenic acid.	632178	Applied for 120 s.	No
				Icon-Dry: 99% ethanol.		Applied for 30 s with air drying.	
3	15			Icon-Infiltrant: resin matrix based on methacrylates, primers and additives.		Applied for 180 s, reapplied for 60 s, cured for 40 s.	Yes
4	15	Clinpro XT Varnish	3 M Unitek (Landsberg, Germany)	Silanized glass powder, silica treated with silicon, methacrylate, 2-hydroxyethyl, water, BIS-GMA, copolymer of acrylic acid and itaconic.	12248	35% phosphoric acid for 20 s, washing and air drying, mixing the components, application and curing for 20 s.	No
5	15						Yes

After white spot lesion treatment, Groups 3 and 5 were submitted to artificial aging. The aging process was achieved by exposure to an ultraviolet irradiation lamp and a tungsten filament mercury vapor atmosphere^27^ with a wavelength of 365 nm, at 45 °C and relative humidity of 65% in a particular machine (Darkroom, Model SL-204, Solab, Piracicaba, Brazil)^28^ for 24 hours, which is equivalent to five years of natural aging.

In sequence, brackets were bonded. Before the bonding procedures, the enamel was polished with a rubber cup and fluoride-free pumice, sprayed with water and then dried with oil-free compressed air stream. Stainless steel brackets for maxillary central incisors (Edgewise standard, slot 0.022 x 0.028-in, 12.25 mm^2^ of area, Morelli, Sorocaba, Brazil) were used. The teeth were etched (35% phosphoric acid gel, 30 seconds), washed with water, and dried by air blowing. Transbond XT system (3M Unitek, Landsberg, Germany) was applied following the manufacturer's instructions. A paper mask was manufactured to standardize the bracket bonding area: a square with 16 mm^2^. The brackets were placed in position with their bases parallel to the floor, pressed firmly against the enamel surface, and excess adhesive was removed with a probe. The samples were light-cured for 60 seconds (15 seconds each from cervical, incisal, mesial and distal directions) by means of Optilux appliance (Demetron Research, Danbury, USA), with a power output of 850 mW/cm^2^, determined by means of a Demetron radiometer (Model 100, Demetron, Danbury, USA). Finally, the specimens were stored in distilled water (37 °C) for 24 hours.

The specimens were placed in a custom-made stabilizing stand to ensure consistency of the point of force application and direction of the debonding force. Subsequently, they were subjected to a shear load test in a universal testing machine (DL10.000, EMIC, São José dos Pinhais, Brazil). A knife-edged shearing rod was used for the test at a crosshead speed of 0.5 mm/min, and a 50 kgf load cell was used for the shear bond strength test.^30^ Force was applied parallel to the buccal surface at the bracket base-enamel interface, and the shear load at the point of failure was recorded in MPa.

Statistical analysis was performed with SPSS 20.0 software (SPSS Inc, Chicago, Ill). Intergroup comparisons were made by one-way analysis of variance (ANOVA) and post hoc Tukey. The level of statistical significance was set at 0.05.

## RESULTS

The results of the shear bond strength tests of brackets bonded to the treated surfaces showed that for specimens in Group 2, treated with Icon, the values were similar to those of the control group. In contrast, specimens in Group 4, treated with Clinpro XT Varnish, showed higher SBS values than those of the control group (*p* < 0.05). The aging process performed in Groups 3 and 5 did not adversely affect the SBS of brackets. Descriptive statistics are presented in Table 2.

**Table 2:** Mean (standard deviation) of shear bond strength (MPa) and ANOVA/Tukey results.

Group	Mean (SD)
1	11.54 (2.31) ^a^
2	10.61(1.82) ^a^
3	12.65 (3.20) ^a^
4	18.60 (4.37) ^b^
5	12.46 (2.79) ^a^

Different letters indicate statistical significance (*p* < 0.05).

## DISCUSSION

In this study, an endeavor was made to simulate the clinical conditions of bonding or rebonding brackets to areas with white spot lesions treated with Clinpro XT Varnish and Icon Infiltrant. An aging process was also performed, so as to simulate material remaining in the mouth for five years and evaluate the bonding procedure to enamel treated for white spots in the past.

Clinpro XT Varnish is a glass ionomer cement that releases fluorine calcium and phosphate, and is widely indicated for treating dentinal hypersensitivity. It is an ionomer sealant that can also be used for preventing^31^ ^,^ ^32^ ^,^ ^33^ and treating^33^ white spot lesions, and is useful for application around orthodontic appliances,^31^ ^,32^ as suggested by the manufacturer. However, this product may not solve the esthetic problem of white spot lesions on the labial surface of teeth. Icon is a very low viscosity resin, with refractive optical properties similar to those of healthy enamel, specifically indicated for cosmetic improvement and interruption of incipient caries. It is known that color stability of the Icon resin lasts for up to 12 months,^34^ and its effectiveness in arresting the progression of noncavitated caries may continue for over three years after application.^35^ Belli et al^24^ applied abrasion tests on Icon infiltrated areas and did not note any differences in vertical wear loss when compared with original enamel.

The results of the shear bond strength tests of brackets bonded to the treated surfaces showed that for specimens in Group 2, treated with Icon, the values were similar to those of the control group. These results are compatible with those obtained by Wiegand et al^21^ and Attin et al.^20^ In contrast, specimens in Group 4, treated with Clinpro XT Varnish, showed higher SBS values than those of the control group (*p* < 0.05). Attin et al^20^ tested another fluoride product (Clinpro White Varnish, 3M Espe, Landsberg, Germany) and found lower values than those found in this study. The difference in the results is probably due to methodological differences. In this study, the tooth surface was cleaned with rubber and prophylactic paste after the application of fluoride varnish and before acid etching. Attin et al^20^ did not perform this step. According to the manufacturer of the product, it can be removed by professional cleaning using pumice. Thus, it is possible that the varnish has been removed wholly or partially from the enamel surface prior to bonding; thus, exposing the previously demineralized surface. Consequently, bond strength increased. It was decided to carry out prophylaxis prior to bonding following the methodology described by a previous study,^36^ and because dentists perform this procedure routinely at the clinical practice before bracket bonding. Kimura et al^36^ studied the effect of fluoride varnish treatment in the bond strength of brackets bonded to enamel using conventional and self-etching adhesives. They found no difference among groups. However, the enamel was not demineralized before fluoride varnish treatment, differing from the present study.

The accelerated artificial aging procedure was applied to Groups 3 and 5 to simulate the clinical condition in which patients had undergone white spot lesion treatment in the past; and later the orthodontist decided to bond a bracket to the infiltrated area. Accelerated aging can change the mechanical properties of resin material and reduce hardness by hydrolysis of the resin matrix. The storage solution usually used is distilled water or artificial saliva, which can infiltrate and reduce frictional forces between the polymer chains, a process known as plasticizing.^37^ However, Groups 3 and 5 presented SBS values close to those of the control group. This indicated that the process of aging infiltrated areas did not adversely affect the SBS of brackets.

The infiltrated surfaces were etched with 35% phosphoric acid before the application of primer and Transbond XT, according to the recommended bonding protocol. One should take into consideration that phosphoric acid has an erosive potential on infiltrants. Thus, etching is able to partially remove the layer of the infiltrated area. However, Hammad et al^38^ showed that the erosion of the infiltrated area exposed to Coke, which contains phosphoric acid, was lower than the erosion caused by natural enamel exposed to the same substance. Nevertheless, the erosion of the infiltrated surface did not appear to be capable of changing the SBS of brackets, because conventional adhesives, such as Transbond XT, are able to penetrate into carious lesions to some extent. Primer monomer formulations with increased TEGDMA (triethylene glycol dimethacrylate) and HEMA (2-hydroxyethyl methacrylate) content have a high penetration capability,^39^ which most likely allows a chemical connection of the resin infiltrant to the primer.^38^ Thus, it is assumed that the primer may also partially penetrate into the demineralized enamel and strengthen the outermost part of the Icon-infiltrated enamel when it is applied after preconditioning.^23^

The results of this study indicate that white spot lesion treatment with the tested dental material did not affect bracket bonding. The results should be extrapolated to clinical practice with caution, as this is a laboratory study. Further clinical researches are suggested to confirm the findings.

## CONCLUSIONS

Treatment for white spots using Icon and Clinpro XT Varnish did not negatively affect the shear strength of orthodontic bonding. Simulated aging of the pretreated area also did not affect the bond strength of brackets.

## References

[1] Moreira TC, Sampaio RKL. (2001). Efeitos do tratamento ortodôntico sobre o esmalte: desmineralização e pigmentação.. Rev Dental Press Ortod Ortop Facial..

[2] Freitas AO, Marquezan M, Nojima MC, Alviano DS, Maia LC. (2014). The influence of orthodontic fixed appliances on the oral microbiota: a systematic review.. Dental Press J Orthod..

[3] Souza-e-Silva CM, Parisotto TM, Steiner-Oliveira C, Kamiya RU, Rodrigues LK, Nobre-dos-Santos M (2013). Carbon dioxide laser and bonding materials reduce enamel demineralization around orthodontic brackets. Lasers Med Sci.

[4] Benson PE, Shah AA, Millett DT, Dyer F, Parkin N, Vine RS (2005). Fluorides, orthodontics and demineralization: a systematic review. J Orthod.

[5] Boersma JG, van der Veen MH, Lagerweij MD, Bokhout B, Prahl-Andersen B. (2005). Caries prevalence measured with QLF after treatment with fixed orthodontic appliances: influencing factors.. Caries Res..

[6] Ahn SJ, Lim BS, Lee YK, Nahm DS. (2006). Quantitative determination of adhesion patterns of cariogenic streptococci to various orthodontic adhesives.. Angle Orthod..

[7] Kerbusch AE, Kuijpers-Jagtman AM, Mulder J, van der Sanden WJ (2010). Prevention of white spots during orthodontic treatment with fixed appliances. Ned Tijdschr Tandheelkd.

[8] Shungin D, Olsson AI, Persson M. (2010). Orthodontic treatment-related white spot lesions: a 14-year prospective quantitative follow-up, including bonding material assessment.. Am J Orthod Dentofacial Orthop..

[9] Tufekci E, Dixon JS, Gunsolley JC, Lindauer SJ (2011). Prevalence of white spot lesions during orthodontic treatment with fixed appliances. Angle Orthod.

[10] Lucchese A, Gherlone E (2013). Prevalence of white-spot lesions before and during orthodontic treatment with fixed appliances. Eur J Orthod.

[11] Julien KC, Buschang PH, Campbell PM (2013). Prevalence of white spot lesion formation during orthodontic treatment. Angle Orthod.

[12] Ten Cate JM, Buijs MJ, Miller CC, Exterkate RAM (2008). Elevated fluoride products enhance remineralization of advanced enamel lesions. J Dent Res.

[13] Cochrane NJ, Cai F, Huq NL, Burrow MF, Reynolds EC (2010). New approaches to enhanced remineralization of tooth enamel. J Dent Res.

[14] Poosti M, Ahrari F, Moosavi H, Najjaran H (2014). The effect of fractional CO2 laser irradiation on remineralization of enamel white spot lesions. Lasers Med Sci.

[15] Paris S, Meyer-Lueckel H, Cölfen H, Kielbassa AM. (2007). Resin infiltration of artificial enamel caries lesions with experimental light curing resins.. Dent Mater J..

[16] Meyer-Lueckel H, Paris S (2008). Improved resin infiltration of natural caries lesions. J Dent Res.

[17] Mueller J, Meyer-Lueckel H, Paris S, Hopfenmuller W, Kielbassa AM. (2006). Inhibition of lesion progression by the penetration of resins in vitro: influence of the application procedure.. Oper Dent..

[18] Ogodescu A, Ogodescu E, Talpos S, Zetu I. (2011). Resin infiltration of white spot lesions during the fixed orthodontic appliance therapy.. Rev Med Chir Soc Med Nat Iasi..

[19] Jimenez EEO, Hilgenberg SP, Rastelli MC, Rsatelli MC, Pilatti GL, Orellana B (2012). Rebonding of unused brackets with different orthodontic adhesives. Dental Press J Orthod..

[20] Attin R, Stawarczyk B, Keçik D, Knösel M, Wiechmann D, Attin T (2012). Shear bond strength of brackets to demineralize enamel after different pretreatment methods. Angle Orthod.

[21] Wiegand A, Stawarczyk B, Kolakovic M, Hämmerle CH, Attin T, Schmidlin PR (2011). Adhesive performance of a caries infiltrant on sound and demineralised enamel. J Dent.

[22] Ekizer A, Zorba YO, Uysal T, Ayrikcila S (2012). Effects of demineralizaton-inhibition procedures on the bond strength of brackets bonded to demineralized enamel surface. Korean J Orthod.

[23] Naidu E, Stawarczyk B, Tawakoli PN, Attin R, Attin T, Wiegand A (2013). Shear bond strength of orthodontic resins after caries infiltrant preconditioning. Angle Orthod.

[24] Belli R, Rahiotis C, Schubert EW, Baratieri LN, Petschelt A, Lohbauer U (2011). Wear and morphology of infiltrated white spot lesions. J Dent.

[25] Queiroz CS, Hara AT, Paes Leme AF, Cury JA (2008). pH-cycling models to evaluate the effect of low fluoride dentifrice on enamel de- and remineralization. Braz Dent J.

[26] Maia LH, L H, Araújo MV, Ruellas AC, Araújo MT. (2012). Incorporation of metal and color alteration of enamel in the presence of orthodontic appliances.. Angle Orthod..

[27] Doray PG, Wang X, Powers JM, Burgess JO. (1997). Accelerated aging affects color stability of provisional restorative materials.. J Prosthodont.

[28] Moreira AD, Mattos CT, de Araújo MV, Ruellas AC, Sant'anna EF (2013). Chromatic analysis of teeth exposed to different mouthrinses. J Dent.

[29] Göhring TN, Zehnder M, Sener B, Schmidlin PR (2004). In vitro microleakage of adhesive-sealed dentin with lactic acid and saliva exposure a radio-isotope analysis. J Dent.

[30] Pithon MM, Oliveira MV, Ruellas AC, Bolognese AM, Romano FL (2007). Shear bond strength of orthodontic brackets to enamel under different surface treatment conditions. J Appl Oral Sci.

[31] Yap J, Walsh LJ, Naser-Ud Din S, Ngo H, Manton DJ (2014). Evaluation of a novel approach in the prevention of white spot lesions around orthodontic brackets. Aust Dent J.

[32] O'Reilly MT, De Jesús Viñas J, Hatch JP (2013). Effectiveness of a sealant compared with no sealant in preventing enamel demineralization in patients with fixed orthodontic appliances a prospective clinical trial. Am J Orthod Dentofacial Orthop.

[33] Kantovitz KR, Pascon FM, Nociti FH, Tabchoury CP, Puppin-Rontani RM (2013). Inhibition of enamel mineral loss by fissure sealant an in situ study. J Dent.

[34] Feng CH, Chu XY (2013). Efficacy of one year treatment of icon infiltration resin on post-orthodontic white spots. Beijing Da Xue Xue Bao.

[35] Meyer-Lueckel H, Bitter K, Paris S (2012). Randomized controlled clinical trial on proximal caries infiltration three-year follow-up. Caries Res.

[36] Kimura T, Dunn WJ, Taloumis LJ (2004). Effect of fluoride varnish on the in vitro bond strength of orthodontic brackets using a self-etching primer system. Am J Orthod Dentofacial Orthop.

[37] Ferracane JL, Berge HX, Condon JR (1998). In vitro aging of dental composites in water--effect of degree of conversion, filler volume, and filler/matrix coupling. J Biomed Mater Res.

[38] Hammad SM, Enan ET. (2013). In vivo effects of two acidic soft drinks on shear bond strength of metal orthodontic brackets with and without resin infiltration treatment.. Angle Orthod.

[39] Paris S, Meyer-Lueckel H, Cölfen H, Kielbassa AM (2007). Penetration coefficients of commercially available and experimental composites intended to infiltrate enamel carious lesions. Dent Mater.

